# Disembodied neurorights? Reconsidering mental integrity and personal identity in memory modification

**DOI:** 10.3389/fpsyg.2025.1731892

**Published:** 2026-01-19

**Authors:** Daniela Guevara, José M. Muñoz

**Affiliations:** 1 Independent Researcher, Valencia, Spain; 2 Head of Neurotechnology, International Center for Neuroscience and Ethics (CINET), Tatiana Foundation, Madrid, Spain

**Keywords:** memory modification, mental integrity, neurorights, neurotechnologies, optogenetics, personal identity, sensorimotor model of memory, somatic markers

*Our bodies are the texts that carry the memories and therefore rememory is no less than reincarnation*.

Katie G. (Cannon, 1993: p. 35)

In a context of rapid economic expansion, different applications of neurotechnology are being researched and developed for use in both medical and non-medical areas, such as employment, education, national defense, and entertainment, among others. Among all these applications, one that is generating increasing ethical and legal debate is the possibility of memory modification using neurotechnologies. In this article, we examine the implications of this type of intervention for the debate on the so-called *neurorights* to mental integrity and personal identity, and argue that the key role played by the body in the formation, consolidation, and retrieval of memories calls for a neurorights debate grounded in integrating the nervous system, the body, the environment, and life history as parts of a unified normative framework. We adopt a conceptual–normative approach aimed at clarifying the content and scope of these two neurorights. While this analysis has regulatory implications, no specific legal instruments for implementation are proposed.

## Memory modification via neurotechnologies: an emergent field

From *Total Recall*^1^ to *Inception*^2^, not forgetting *Eternal Sunshine of the Spotless Mind*^3^, cinema and literature have often fantasized about the possibility of modifying memories in various ways. *Memory modification*, as it will be understood in this article, encompasses a wide range of possibilities, including: (a) enhancing or diminishing the ability to remember; (b) implanting or erasing complete memories; (c) implanting, erasing, or altering fragments of memories; (d) intervening on memory reconsolidation; and (e) adjusting or transforming the emotional valence of memories (i.e., their pleasant, unpleasant, or neutral “tone”).

Nonetheless, it is not necessary to resort to fiction to modify memories, which is, in fact, already possible through various current *neurotechnologies*; some examples being the following:

- Non-invasive brain stimulation for treating post-traumatic stress disorder and other neurological and psychiatric conditions (Farina and Lavazza, 2022). This includes neurofeedback, which “can modulate brain activity via real-time monitoring and feedback of EEG or fMRI signals” (Chiba et al., 2019: p. 2; see also Taschereau-Dumouchel et al., 2021).
- Transcranial magnetic stimulation (TMS) for preventing the reconsolidation of fear memories (Borgomaneri et al., 2020).
- Reduction of fear responses (e.g., arachnophobia) by means of neuropharmaceuticals (Soeter and Kindt, 2015).

A cutting-edge neurotechnology that seems especially promising for memory modification is *optogenetics*, which consists of the convergence of genetic engineering and brain stimulation. As explained by Adamczyk and Zawadzki (2020: p. 207–208),

neurons of interest are genetically modified to be made responsive to light, which is done by means of inserting opsin genes (genes that express light-sensitive proteins). […] Once opsin genes arrive at their determined destination, they cause neurons to express light-sensitive proteins. When illuminated with light, the channels of these proteins regulate the flow of electrically charged ions across membranes, exacerbating or inhibiting the neuron's firing of action potentials, thus facilitating or preventing its communication with other neurons (depending on which light-activated protein is used). Thanks to this procedure, specific neurons can be activated or deactivated “at will,” making optogenetics a highly selective and precise technique for manipulating neural activity.

Thus, by acting on target neural circuits that have been previously identified and genetically modified, optogenetics makes it possible to intervene selectively in the brain, which would in principle allow for memory modification with unprecedented precision and effectiveness. Despite this promise, optogenetic studies have so far been conducted mainly on rodents (e.g., Liu et al., 2014; Etter et al., 2019; Davis and Vanderheyden, 2020) and non-human primates (e.g., Stauffer et al., 2016; Deng et al., 2018), with only isolated clinical cases in humans (Sahel et al., 2021), which calls for prudence at this time. Nonetheless, rodent memory can now be manipulated via optogenetics to treat post-traumatic memories (Davis and Vanderheyden, 2020), retrieve spatial memory (Etter et al., 2019), and even create false memories (Liu et al., 2014). While the applicability of these findings to humans remains uncertain, their potential ethical, social, and legal implications can hardly be overlooked.

## Ethical considerations: impact of memory modification on integrity and identity

*Prima facie*, memory modification may provide substantial clinical benefits—including the treatment of trauma, amnesia, and some forms of dementia—and might enhance the cooperation of victims and witnesses in criminal investigations. However, it also carries significant risks at the mental level.^4^ For example, since intervention with optogenetics requires the (irreversible) genetic manipulation of neurons, the integrity of neural circuits could be compromised. Moreover, as highlighted by Zawadzki and Adamczyk (2021: p. 897), “[r]emoval or emotional modification of difficult memories may result in diminishing a patient's sense of agency, that is, her feelings and beliefs about her ability to control herself, the world, and others, and to make her own decisions.” These authors also emphasize the potential impact of memory modification on personal growth and narrative identity:

[I]f an individual were to decide to remove (with the use of optogenetics) or to change or dampen the emotional component (with the use of optogenetics, propranolol, or PFC–rTMS) of negative memories, she might deprive herself of the opportunity to experience a redemption sequence. Thus, such interventions might deprive an individual of the potential for finding meaning in suffering and prevent her from reshaping emotionally negative events into positive outcomes such as self-transformation, or involvement in prosocial goals, for example, helping others. […] The redemption sequence points to the broader adaptational problem of how humans make sense of suffering in their lives in narrative terms Zawadzki and Adamczyk, (2021): p. 894–895).

Thus, while memory manipulation via neurotechnologies has the potential to improve the quality of life for some individuals, it simultaneously entails significant challenges related to two key ethical principles: personal identity and mental integrity. Importantly, both principles have recently been proposed as components of a human rights framework of so-called neurorights, which are “the ethical, legal, social, or natural principles of freedom or entitlement related to a person's cerebral and mental domain” Ienca, (2021): p. 1).

According to (Muñoz 2025), “the concept of *mental integrity* (MI) […] refers to a possible moral and/or legal right for the protection of the intactness of the mind” and, as explained by Ienca and Andorno (2017: p. 18), it “should not only guarantee the right of individuals with mental conditions to access mental health schemes and receive psychiatric treatment or support wherever needed,” but also “the right of all individuals to protect their mental dimension from potential harm.” Therefore, given that the insertion of opsin genes into neural circuits is irreversible, any optogenetic memory manipulation should be carefully examined to ensure it aligns with human rights, guaranteeing that the individual does not suffer harm to the intactness of their mental sphere. Memory manipulation through other neurotechnologies (e.g., neurofeedback, TMS, neuropharmaceuticals) should also adhere to the same safeguard parameters to be respectful with the neuroright to mental integrity.

*Personal identity*, in turn, has been proposed as a neuroright by the Neurorights Foundation^5^ and, under the designation of “psychological continuity,” by Ienca and Andorno (2017: p. 20), who characterize it as “the crucial requirement of personal identity consisting in experiencing oneself as persisting through time as the same person.” Again, for memory manipulation by means of neurotechnologies to align with a human rights framework, it must provide guarantees that it will not result in psychological discontinuity incompatible with the preservation of essential characteristics of the individual's identity—such as their personal growth or processes of redemption and self-transformation. In Ienca and Andorno's words, “memory engineering technologies may impact a person's identity by selectively removing, altering, adding or replacing individual memories that are relevant to their self-recognition as persons” (Ienca and Andorno, 2017: p. 20).

## The importance of the body in memory formation and consolidation

In the previous section, the consequences of memory manipulation via neurotechnologies that do not respect the integrity and identity of the person were considered primarily from a brain-centered perspective. Nonetheless, while the prominence of the brain in memory formation, consolidation, and retrieval cannot be denied nor underestimated, there is growing evidence that the body also plays a key role through mechanisms as diverse as gestures Macedonia, (2014); Cook et al., (2017), smiling (Arminjon et al., 2015), exercise (Snigdha et al., 2014), secretion of adrenal hormones (McGaugh, 2013), gut microbiota activity (Arnoriaga-Rodríguez et al., 2020), cardiac interoception (Werner et al., 2010), and control of respiration (Zelano et al., 2016; Arshamian et al., 2018; Heck et al., 2019; Schreiner et al., 2023). This evidence reinforces theories of embodied and enactive cognition. According to the former, “the body's neural and extraneural processes, as well as its mode of coupling with the environment, play important roles in cognition” (Gallagher, 2023: p. 1), while the latter “emphasize the idea that perception is *for action*, and that this action orientation shapes most cognitive processes” (Gallagher, 2023: p. 30).

The importance of the body in memory has also been an important focus of the highly influential book *Descartes' Error*, in which (Damasio 1994) emphasizes that considering the connection between the body and the mind is essential for understanding perception and information processing. He proposes that so-called *somatic markers*—bodily state changes associated with emotional experiences—are instrumental in decision-making and information processing (see also Muñoz, 2017). These markers can be positive or negative and are stored as patterns of neural activity linked to past experiences, influencing how memories are encoded and retrieved. They also serve to evaluate alternatives and predict outcomes:

In short, *somatic markers are a special instance of feelings generated from secondary emotions*. Those emotions and feelings *have been connected, by learning, to predicted future outcomes of certain scenarios*. When a negative somatic marker is juxtaposed to a particular future outcome the combination functions as an alarm bell. When a positive somatic marker is juxtaposed instead, it becomes a beacon of incentive Damasio, (1994): p. 174).

In another influential book called *The Body Keeps the Score*, (Van der Kolk 2014) argues that memory is not limited to the mental representation of past events. According to his approach, which is based on the study of traumatic experiences, so-called bodily memory allows the formation of memories in the form of movement patterns, posture, and muscle tension. In this way, memories are stored not only in the mind but also in the body, and they can be reactivated through sensory and motor experiences.^6^

## Neurorights as embodied rights

The connection between the body and the brain is bidirectional. As mentioned, the brain processes sensory and emotional information, while the body stores and expresses memories through patterns of movement and posture, as well as physiological and metabolic processes. Communication between the body and the brain is essential for memory; as explained by (LeDoux J 2000: p. 177) for the case of working memory:

Although the amygdala does not have extensive connections with the dorsolateral prefrontal cortex, it does communicate with the anterior cingulate and orbital cortex, two other components of the working memory network. But in addition, the amygdala projects to non-specific systems involved in the regulation of cortical arousal and it controls bodily responses (behavioral, autonomic, endocrine), which then provide feedback that can influence cortical processing indirectly.

Moreover, memory retrieval would not only require accessing cognitive information but also the bodily and emotional experience associated with the original event. This way of understanding memory, referred to by some researchers as the *sensorimotor model of memory*, “predicts that memory processes can be manipulated through manipulation of the body” (Ianì, (2019): p. 1,747).

Currently, neurotechnologies are not yet at a stage of development that allows for advanced or highly precise memory modification. However, if they ever reach that point—and if the sensorimotor model of memory proves to be correct—it would become urgent to thoroughly examine the potential clinical risks arising from a possible decoupling between the brain and the body as a result of memory modification through the nervous system alone. For example, from a perspective not related to treatment but rather to memory enhancement in healthy individuals, so-called neuroenhancement—which involves intervention exclusively in the nervous system (Clark and Parasuraman, 2014)—would not necessarily lead to an improvement of *the individual*—in addition to other significant concerns (see Muñoz and Borbón, 2023).

The distinction between therapeutic and non-therapeutic interventions is normatively significant. The principle of proportionality applies differently in each case, since the potential benefits of therapeutic interventions can, in principle, justify a higher tolerance for risk. This normative difference affects the setting of regulatory thresholds that separate permissible from impermissible interventions and entails different designs for consent standards across the two types of intervention. An embodied view of mental integrity and personal identity has direct implications for how these ethical and regulatory principles and standards are designed and applied.

From an ethical standpoint, it is both compatible and desirable to integrate the conception of memory as a brain–body mechanism into a normative framework that already, *de facto*, understands integrity and identity as principles applied to the whole individual. The right to mental integrity is a good example in this regard, as illustrated by Ienca and Andorno's description of the inseparability between mental and bodily integrity within the European legal framework:

The right to personal physical and mental integrity is protected by the EU's Charter of fundamental rights (Article 3), stating that “everyone has the right to respect for his or her physical and mental integrity.” Understandably, the Charter emphasizes the importance of this right in the fields of medicine and biology, because of the direct impact that biomedical technologies may have on people's physical and mental integrity (Ienca and Andorno, 2017: p. 18).

The central role of the brain and the rest of the nervous system in the formation, consolidation, and retrieval of memories is undeniable. Nonetheless, research on memory modification must not overlook bodily role, interactions with the environment, and individual life history. Legal frameworks for the protection of rights related to the human brain and mind (i.e., neurorights) should avoid neuroessentialist (Reiner, 2011) and brain-in-a-vat (Hickey, n.d.) mindsets and be based on this holistic perspective to ensure comprehensive protection of the individual against potential misuse of neurotechnologies. A person is a complex being shaped by a wide range of biological (e.g., the brain), mental, and social components, and it is the whole person who must be the object of legal protection. As beautifully put by Damasio (1994: p. xvii): “The soul breathes through the body, and suffering, whether it starts in the skin or in a mental image, happens in the flesh.”

## Footnotes

1 See the profile of this film at https://www.filmaffinity.com/us/film992611.html.

2 See the profile of this film at https://www.filmaffinity.com/us/film971380.html.

3 See the profile of this film at https://www.filmaffinity.com/us/film982810.html.

4 Since this article focuses on the effects of memory modification on identity and integrity, other effects will not be addressed here. Analyses of these additional effects can be found in (Adamczyk and Zawadzki 2020) and (González-Márquez 2023).

5 See https://www.neurorightsfoundation.org/mission/impact.

6 In contrast with Damasio's and van der Kolk's views, computational and representational accounts tend to locate cognition and memory primarily at the level of neural information processing, treating the body as a peripheral or non-constitutive component (see Rescorla, 2024).

## References

[B1] Adamczyk A. K. Zawadzki P. (2020). The memory-modifying potential of optogenetics and the need for neuroethics. Nanoethics 14, 207–225. doi: 10.1007/s11569-020-00377-1

[B2] Arminjon M. Preissmann D. Chmetz F. Duraku A. Ansermet F. Magistretti P. J. . (2015). Embodied memory: unconscious smiling modulates emotional evaluation of episodic memories. Front. Psychol. 6:650. doi: 10.3389/fpsyg.2015.00650 26074833 PMC4443770

[B3] Arnoriaga-Rodríguez M. Mayneris-Perxachs J. Burokas A. Contreras-Rodríguez O. Blasco G. Coll C. . (2020). Obesity impairs short-term and working memory through gut microbial metabolism of aromatic amino acids. Cell Metab. 32, 548–560.e7. doi: 10.1016/j.cmet.2020.09.002 33027674

[B4] Arshamian A. Iravani B. Majid A. Lundström J. N. (2018). Respiration modulates olfactory memory consolidation in humans. J. Neurosci. 38, 10286–10294. doi: 10.1523/JNEUROSCI.3360-17.2018 30348674 PMC6596216

[B5] Borgomaneri S. Battaglia S. Garofalo S. Tortora F. Avenanti A. di Pellegrino G. . (2020). State-dependent TMS over prefrontal cortex disrupts fear-memory reconsolidation and prevents the return of fear. Curr. Biol. 30, 3672–3679.e4. doi: 10.1016/j.cub.2020.06.091 32735813

[B6] Cannon K. G. (1993). Womanist perspectival discourse and cannon formation. J. Femin. Stud. Relig. 9, 29–37.

[B7] Chiba T. Kanazawa T. Koizumi A. Ide K. Taschereau-Dumouchel V. Boku S. . (2019). Current status of neurofeedback for post-traumatic stress disorder: a systematic review and the possibility of decoded neurofeedback. Front. Hum. Neurosci. 13:233. doi: 10.3389/fnhum.2019.00233 31379538 PMC6650780

[B8] Clark V. P. Parasuraman R. (2014). Neuroenhancement: enhancing brain and mind in health and in disease. Neuroimage 85, 889–894. doi: 10.1016/j.neuroimage.2013.08.071 24036352

[B9] Cook S. W. Friedman H. S. Duggan K. A. Cui J. Popescu V. (2017). Hand gesture and mathematics learning: lessons from an avatar. Cogn. Sci. 41, 518–535. doi: 10.1111/cogs.12344 27128822

[B10] Damasio A. R. (1994). Descartes' error, Avon Books.

[B11] Davis C. J. Vanderheyden W. M. (2020). Optogenetic sleep enhancement improves fear-associated memory processing following trauma exposure in rats. Sci. Rep. 10:18025. doi: 10.1038/s41598-020-75237-9 33093538 PMC7581760

[B12] Deng C. Yuan H. Dai J. (2018). Behavioral manipulation by optogenetics in the nonhuman primate. Neuroscientist 24, 526–539. doi: 10.1177/1073858417728459 28874078

[B13] Etter G. van der Veldt S. Manseau F. Zarrinkoub I. Trillaud-Doppia E. Williams S. . (2019). Optogenetic gamma stimulation rescues memory impairments in an Alzheimer's disease mouse model. Nat. Commun. 10:5322. doi: 10.1038/s41467-019-13260-9 31757962 PMC6876640

[B14] Farina M. Lavazza A. (2022). Memory modulation via non-invasive brain stimulation: status, perspectives, and ethical issues. Front. Hum. Neurosci. 16:826862. doi: 10.3389/fnhum.2022.826862 35308617 PMC8931830

[B15] Gallagher S. (2023). Embodied and Enactive Approaches to Cognition. Cambridge, UK: Cambridge University Press. doi: 10.1017/9781009209793

[B16] González-Márquez C. (2023). Neuromodulation and memory: exploring ethical ramifications in memory modification treatment via implantable neurotechnologies. Front. Psychol. 14:1282634. doi: 10.3389/fpsyg.2023.1282634 38179489 PMC10764565

[B17] Heck D. H. Kozma R. Kay L. M. (2019). The rhythm of memory: how breathing shapes memory function. J. Neurophysiol. 122, 563–571. doi: 10.1152/jn.00200.2019 31215344 PMC6734396

[B18] Hickey L. P. (n.d.). “The brain in a vat argument,” in Internet Encyclopedia of Philosophy, eds. J. Fieser J and B. Dowden. Available online at: https://iep.utm.edu/brain-in-a-vat-argument/ (Accessed January 7, 2026).

[B19] Ianì F. (2019). Embodied memories: reviewing the role of the body in memory processes. Psychon. Bull. Rev. 26, 1747–1766. doi: 10.3758/s13423-019-01674-x 31654375

[B20] Ienca M. (2021). On neurorights. Front. Hum. Neurosci. 15:701258. doi: 10.3389/fnhum.2021.701258 34630057 PMC8498568

[B21] Ienca M. Andorno R. (2017). Towards new human rights in the age of neuroscience and neurotechnology. Life Sci. Soc. Policy 13:5. doi: 10.1186/s40504-017-0050-1 28444626 PMC5447561

[B22] LeDoux J. E. (2000). Emotion circuits in the brain. Annu. Rev. Neurosci. 23, 155–184. doi: 10.1146/annurev.neuro.23.1.155 10845062

[B23] Liu X. Ramirez S. Tonegawa S. (2014). Inception of a false memory by optogenetic manipulation of a hippocampal memory engram. Philos. Trans. R. Soc. B 369:20130142. doi: 10.1098/rstb.2013.0142 24298144 PMC3843874

[B24] Macedonia M. (2014). Bringing back the body into the mind: gestures enhance word learning in foreign language. Front. Psychol. 5:1467. doi: 10.3389/fpsyg.2014.01467 25538671 PMC4260465

[B25] McGaugh J. L. (2013). Making lasting memories: remembering the significant. Proc. Natl. Acad. Sci. U. S. A. 110, 10402–10407. doi: 10.1073/pnas.1301209110 23754441 PMC3690616

[B26] Muñoz J. M. (2017). Somatic markers, rhetoric, and post-truth. Front. Psychol. 8:1273. doi: 10.3389/fpsyg.2017.01273 28798708 PMC5526910

[B27] Muñoz J. M. (2025). “Mental integrity,” in The International Encyclopedia of Ethics, ed. H. LaFollette (Blackwell). doi: 10.1002/9781444367072.wbiee990

[B28] Muñoz J. M. Borbón D. (2023). Equal access to mental augmentation: should it be a fundamental right? Brain Stimul. 16, 1094–1096. doi: 10.1016/j.brs.2023.05.003 37268292

[B29] Reiner P. B. (2011). “The rise of neuroessentialism,” in The Oxford Handbook of Neuroethics, eds. J. Illes and B. J. Sahakian (Oxford, UK: Oxford University Press). doi: 10.1093/oxfordhb/9780199570706.013.0049

[B30] Rescorla M. (2024). “The computational theory of mind,” in Stanford Encyclopedia of Philosophy, eds. E. N. Zalta and U. Nodelman. Available online at: https://plato.stanford.edu/archives/fall2025/entries/computational-mind (Accessed January 7, 2026).

[B31] Sahel J. A. Boulanger-Scemama E. Pagot C. Arleo A. Galluppi F. Martel J. N. . (2021). Partial recovery of visual function in a blind patient after optogenetic therapy. Nat. Med. 27, 1223–1229. doi: 10.1038/s41591-021-01351-4 34031601

[B32] Schreiner T. Petzka M. Staudigl T. Staresina B. P. (2023). Respiration modulates sleep oscillations and memory reactivation in humans. Nat. Commun. 14:8351. doi: 10.1038/s41467-023-43450-5 38110418 PMC10728072

[B33] Snigdha S. de Rivera C. Milgram N. W. Cotman C. W. (2014). Exercise enhances memory consolidation in the aging brain. Front. Aging Neurosci. 6:3. doi: 10.3389/fnagi.2014.00003 24550824 PMC3910002

[B34] Soeter M. Kindt M. (2015). An abrupt transformation of phobic behavior after a post-retrieval amnesic agent. Biol. Psychiatry 78, 880–886. doi: 10.1016/j.biopsych.2015.04.006 25980916

[B35] Stauffer W. R. Lak A. Yang A. Borel M. Paulsen O. Boyden E. S. . (2016). Dopamine neuron-specific optogenetic stimulation in rhesus macaques. Cell 166, 1564–1571.e6. doi: 10.1016/j.cell.2016.08.024 27610576 PMC5018252

[B36] Taschereau-Dumouchel V. Cortese A. Lau H. Kawato M. (2021). Conducting decoded neurofeedback studies. Soc. Cogn. Affect. Neurosci. 16, 838–848. doi: 10.1093/scan/nsaa063 32367138 PMC8343564

[B37] Van der Kolk B. (2014). The Body Keeps the Score. New York, NY: Penguin.

[B38] Werner N. S. Peres I. Duschek S. Schandry R. (2010). Implicit memory for emotional words is modulated by cardiac perception. Biol. Psychol. 85, 370–376. doi: 10.1016/j.biopsycho.2010.08.008 20813152

[B39] Zawadzki P. Adamczyk A. K. (2021). To remember, or not to remember? Potential impact of memory modification on narrative identity, personal agency, mental health, and well-being. Bioethics 35, 891–899. doi: 10.1111/bioe.12926 34427951 PMC9291322

[B40] Zelano C. Jiang H. Zhou G. Arora N. Schuele S. Rosenow J. . (2016). Nasal respiration entrains human limbic oscillations and modulates cognitive function. J. Neurosci. 36, 12448–12467. doi: 10.1523/JNEUROSCI.2586-16.2016 27927961 PMC5148230

